# MicroRNA-34a is a potent tumor suppressor molecule *in vivo *in neuroblastoma

**DOI:** 10.1186/1471-2407-11-33

**Published:** 2011-01-25

**Authors:** Amanda Tivnan, Lorraine Tracey, Patrick G Buckley, Leah C Alcock, Andrew M Davidoff, Raymond L Stallings

**Affiliations:** 1 Department of Cancer Genetics, Royal College of Surgeons in Ireland, York House, York Street, Dublin 2, Ireland; 2 National Children's Research Centre, Our Lady's Children's Hospital, Crumlin, Dublin 12, Ireland; 3 Department of Surgery, St. Jude Children's Research Hospital, Memphis, TN 38105, USA; 4 Department of Surgery, University of Tennessee Health Science Center, Memphis, TN 38105, USA

## Abstract

**Background:**

Neuroblastoma is a paediatric cancer which originates from precursor cells of the sympathetic nervous system and accounts for 15% of childhood cancer mortalities. With regards to the role of miRNAs in neuroblastoma, miR-34a, mapping to a chromosome 1p36 region that is commonly deleted, has been found to act as a tumor suppressor through targeting of numerous genes associated with cell proliferation and apoptosis.

**Methods:**

A synthetic miR-34a (or negative control) precursor molecule was transfected into NB1691^luc ^and SK-N-AS^luc ^neuroblastoma cells. Quantitative PCR was used to verify increased miR-34a levels in NB1691^luc ^and SK-N-AS^luc ^cell lines prior to *in vitro *and *in vivo *analysis. *In vitro *analysis of the effects of miR-34a over expression on cell growth, cell cycle and phosphoprotein activation in signal transduction pathways was performed. Neuroblastoma cells over expressing miR-34a were injected retroperitoneally into immunocompromised CB17-SCID mice and tumor burden was assessed over a 21 day period by measuring bioluminescence (photons/sec/cm^2^).

**Results:**

Over expression of miR-34a in both NB1691^luc ^and SK-N-AS^luc ^neuroblastoma cell lines led to a significant decrease in cell number relative to premiR-negative control treated cells over a 72 hour period. Flow cytometry results indicated that miR-34a induced cell cycle arrest and subsequent apoptosis activation. Phosphoprotein analysis highlighted key elements involved in signal transduction, whose activation was dysregulated as a result of miR-34a introduction into cells. As a potential mechanism of miR-34a action on phosphoprotein levels, we demonstrate that miR-34a over-expression results in a significant reduction of *MAP3K9 *mRNA and protein levels. Although *MAP3K9 *is a predicted target of miR-34a, direct targeting could not be validated with luciferase reporter assays. Despite this fact, any functional effects of reduced MAP3K9 expression as a result of miR-34a would be expected to be similar regardless of the mechanism involved. Most notably, *in vivo *studies showed that tumor growth was significantly repressed after exogenous miR-34a administration in retroperitoneal neuroblastoma tumors.

**Conclusion:**

We demonstrate for the first time that miR-34a significantly reduces tumor growth in an *in vivo *orthotopic murine model of neuroblastoma and identified novel effects that miR-34a has on phospho-activation of key proteins involved with apoptosis.

## Background

MicroRNAs (miRNAs) are noncoding RNA molecules which act as post-transcriptional regulators of specific messenger RNA transcripts (mRNAs), resulting in targeted degradation and suppression of gene expression. MiRNAs play major roles in normal developmental processes [1,2], and their dysregulation significantly contributes to various aspects of carcinogenesis in nearly all forms of cancer, negatively regulating both tumor suppressor and oncogenes [3]. As reviewed by Stallings *et al*., miRNAs play particularly important roles in the pathogenesis of neuroblastoma, a paediatric cancer originating from precursor cells of the sympathetic nervous system [4,5]. Neuroblastomas are particularly problematic in that some genetic subtypes, such as those exhibiting amplification of the *MYCN *oncogene or deletion of chromosome 11q, are associated with very poor patient survival in spite of intensive multimodal chemotherapy.

MiR-34a maps to the distal region of chromosome 1p which is commonly deleted in neuroblastoma and was first identified as having a tumor suppressive function in neuroblastoma [6]. Tumors with loss of 1p are more commonly of the *MYCN *amplified variety (see Stallings for review [7]). Specifically, in the study by Welch *et al*., [6] and in later studies [8,9], ectopic over-expression of miR-34a in neuroblastoma cell lines resulted in the activation of a caspase-mediated apoptotic pathway. The importance of miR-34a in cancer is now firmly established, having tumor suppressive effects in multiple types of cancer, including leukemias [10], hepatocellular carcinoma [11], pancreatic [12] and colon [13], among others. MiR-34a has multiple experimentally validated targets involved with cellular proliferation and apoptosis, such as *MYCN*, *BCL2*, *SIRT1, SFRP1 CAMTA1*, *NOTCH1*, *JAG1*, *CCND1*, *CDK6 *and *E2F3 *[6,9,14-19]. Notably, miR-34a is directly up-regulated by p53 [14,20-22] and a related family member, miR-34c, also has tumor suppressive affects [8,23,24].

Although the direct effects of miR-34a over-expression have been studied in a wide range of cancer cells *in vitro*, relatively few *in vivo *studies involving miR-34a have been reported. Transient transfection of glioblastoma cells with synthetic miR-34a subsequently affected tumor growth in a murine xenograft model [25]. Additionally, the *in vivo *tumor suppressive effects of miR-34a were noted in a human xenograft model of colon cancer as a result of tumor-site administration of solubilised synthetic miR-34a [13] and in a xenograft model of lung cancer [26,27]. It was therefore of interest to evaluate the role of miR-34a *in vivo *in an orthotopic murine model of neuroblastoma through assessment of tumor growth and moribundity relative to control miRNA treated cohorts.

Although, as previously mentioned, a number of miR-34a direct targets have been identified, the down-stream effects of miR-34a are still poorly understood. Chang *et al*., carried out Affymetrix gene expression profiling in HCT116 pancreatic cancer cells with, and without, miR-34a over expression [20]. Interestingly, the presence of miR-34a resulted in the up-regulation of 532 mRNA transcripts and the corresponding reduction in 681 mRNA transcripts. Gene transcripts which were down regulated showed significant enrichment of miR-34a binding sites in the 3'UTR; however, the additional increase in gene expression indicates the broad range of genes which are affected, downstream of direct target binding, which might be involved in miR-34a-induced apoptosis. Identification of varied transcript expression of several genes involved in MAPK signalling in pancreatic cancer cells [20], lung cancer cells [27] and also the identification of *MAP3K9 *as a predicted target of miR-34a, led to our interest in the relevance of alterations in activated phosphoproteins involved in signal transduction, in response to miR-34a over expression.

## Methods

### Cell Lines and Transfection Experiments

Kelly, NB1691^luc ^and SK-N-AS^luc ^cell lines were maintained in RPMI-1640 supplemented with heat-inactivated foetal bovine serum (10%), l-glutamine (1%) and 100 μg/mL Zeocin (InVivoGen, San Diego, California). Although Kelly and SK-N-AS cell lines have been well characterized by aCGH for DNA copy number alterations, neither the NB1691^luc ^line, nor the luciferase expressing subline of SK-N-AS, have ever been characterized by aCGH. aCGH analysis of all four cell lines used in this study is detailed in Additional File 1, Figure S1a-d. Kelly and SK-N-AS lines had all of the genomic imbalances identified in prior publications, while the SK-N-AS^luc ^line was identical in all respects to the parental SK-N-AS line. NB1691^luc ^exhibited MYCN and MDM2 amplification, as previously noted [28].

The Pre-miR™ to miR-34a (30 μM) and the premiR-negative control miRNA (negative control 1, Applied Biosystems) were reverse transfected into NB1691^luc ^and SK-N-AS^luc ^cell lines using the transfection agent siPORT™ NeoFX™ (Applied Biosystems/Ambion, Austin, TX). Cell culture-transfection media was changed after 24 hours and replaced with pre-warmed standard cell culture media. In experiments where qPCR was intended for analysis, total RNA/miRNA was extracted 48 hours post-transfection using RNeasy Kit/miRNeasy^© ^kit (Qiagen Inc, Valencia, CA).

### Reverse transcription and Real-time qPCR

Reverse transcription was carried out using 50 ng of total RNA with a primer specific for miR-34a and the TaqMan microRNA reverse transcription kit (Applied Biosystems). Quantitative PCR (qPCR) was carried out on the 7900 HT Fast Real-time System (Applied Biosystems). RNU66, a small RNA encoded in the intron of *RPL5 *(chr1:93,018,360-93,018,429; 1p22.1), was used for normalization in miRNA studies and β-actin was used for normalization in gene expression studies. A relative fold change in expression of the target miRNA/gene transcript was determined using the comparative cycle threshold method (2^-ΔΔCT^).

### Western Blotting

Total protein was isolated from cells using a radioimmunoprecipitation assay (RIPA) lysis buffer (Sigma-Aldrich Corp., St. Louis, MO). Protein concentration was measured using the BCA assay from Millipore (Millipore Corp., Billerica, USA). Proteins were fractionated on 10% polyacrylamide gels, and blotted onto nitrocellulose membrane. The membrane was probed with the Anti-MAP3K9 C-terminal antibody (Abcam, Cambridge, MA #ab71628) and anti β-Actin (Abcam, Cambridge, MA #ab8226 ) was used as a loading control. Signal was detected using Immoblion Western (Millipore Corp., Billerica, USA).

### Luciferase reporter assays

A 1,140 bp region of the 3'UTR of *MAP3K9 *containing the predicted miR-34a binding site was inserted into the dual luciferase PsiCheck2 reporter vector (Promega), designated Psi/miR-34a (Additional File 2, Figure S2a). As a negative control, a 5 nt mutation was introduced into the miR-34a seed region of this sequence, designated Psi/miR-34amut (Additional File 2, Figure S2b). Kelly, NB1691 or SK-N-AS cells were plated in 6 well format, and co-transfected using Lipofectamine 2000 with either 2 μg of Psi/miR-34a or Psi/miR-34amut, along with either 30 nm of either the Pre-miR-34a or a scrambled oligonucleotide negative control. Luciferase activity was measured after 24, 48 and 72 hours using a Viktor Plate Reader (Additional File 3, Figure S3A-F)

### Growth Curve and Cell cycle analysis

*In vitro *experiments were carried out in triplicate in 6 well plates. Neuroblastoma cells (3 × 10^5^) were reverse transfected with premiR-34a or premiR-negative control and an additional set of wells remained untreated (non-transfected). At the appropriate time point, 24, 48 and 72 hours post-transfection, cells were trypsinised and re-suspended in 1 ml of media and nuclei were counted in triplicate for each sample using a Beckman Coulter Cell counter (Beckman Coulter Inc, Brea, CA). In additional experiments, NB1691^luc ^cells were isolated 48 and 72 hours post-transfection and analysed by flow cytometry for DNA content (cell cycle) and Annexin-V staining (apoptosis, n = 3). Fold change in cell cycle and apoptosis, at each time point, was calculated relative to premiR-negative control-treated samples.

### Multi-Pathway Signalling Phosphoprotein analysis

NB1691^luc ^cells were reverse transfected with premiR-34a and cell pellets were isolated 48 hours post-transfection and treated with lysis buffer (Millipore Corp., Billerica, USA) containing phosphatise and protease inhibitors. Cell debris was removed by centrifugation at 14 000 g for 15 min. Equal amounts of soluble protein lysates were analysed for the level of various phosphorylated proteins using MILLIPLEX MAP 8-plex Multi-Pathway Signalling Phosphoprotein kit on the Luminex 200 system (Millipore Corp.).

### *In vivo* tumor establishment and imaging

All animal experiments were carried out in 4 week old CB-17/SCID mice (Charles' River Laboratories, Wilmington, MA) and were performed in accordance with a protocol approved by the Institutional Animal Care and Use Committee of St Jude Children's Research Hospital, Memphis, Tennessee. Retroperitoneal tumors were established by injection of 4.4 × 10^5 ^NB1691^luc ^or SK-N-AS^luc ^cells behind the left adrenal gland via a left subcostal incision during administration of isoflurane (2%). Mice received an intraperitoneal injection of D-Luciferin (150-mg/kg, Caliper Life Sciences, Hopkinton, MA) and, five minutes after substrate injection, *in vivo *bioluminescence images were obtained using an IVIS Imaging System 100 Series (Xenogen Corporation, Alameda, CA). All specimens were imaged at a range of 25 cm and acquired images were analyzed using Living Image Software version 2.5 (Xenogen). *In vivo *bioluminescence measurements were recorded as photons per second and the automatic range of interest function of the Living Image Software was used to analyze tumor bioluminescence in the retroperitoneal tumors resulting in a value of photons per second per centimetre squared (photons/sec/cm^2^). Mice were initially imaged for 1 minute and if an image were saturated, the image time was reduced by 10-second intervals until saturation was eliminated.

### Statistical analysis

Bioluminescence intensities are reported as the mean photons/sec/cm^2^± SEM. The GraphPad Prism program (Prism 5, GraphPad Software Inc., La Jolla, CA) was used to analyze and graphically present all *in vitro *and *in vivo *data. Two-Way ANOVA analysis was used to analyze significance of cell line growth curves, mi-RNA expression by qPCR and tumor bioluminescence over time. A t-test was used to compare cell cycle distribution, apoptosis induction and phosphoprotein activation. Mantle-Cox analysis was used to compare overall survival in xenograft cohorts and Wilcoxon Rank Sum Test was carried out on qPCR expression data for *MAP3K9 *mRNA transcripts.

## Results

Although the phenotypic effects of miR-34a over-expression have been extensively investigated in a number of neuroblastoma cell lines, the impact of miR-34a on the *in vivo *growth of neuroblastoma tumors using an orthotopic mouse model has never been investigated. In order to further our understanding of the effects of miR-34a as a potential tumor suppressor, we have carried out transfection studies of this miRNA in the context of a well characterized orthotopic mouse model of this disease [29]. Two cell lines, both containing a stable, constitutively expressed luciferase reporter construct for measuring tumor growth were used, NB1691^luc ^(*MYCN *amplified) and SK-N-AS^luc ^(non *MYCN *amplified).

**Figure 1:**
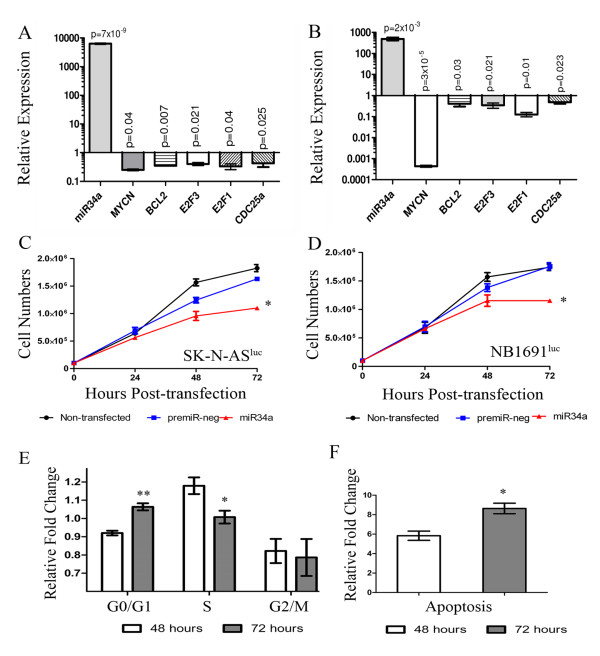
**Growth curves and cell cycle analysis**. SK-N-AS^luc ^and NB1691^luc ^(1 × 10^6^) cells were reverse transfected with premiR-34a (30 μM) or a premiR-negative control molecule and cell pellets were analysed after 48 hours by qPCR for miR-34a, *MYCN*, *BCL2, E2F1, E2F3 and CDC25A *levels (Figure 1A and B, respectively). Additionally, cells were isolated at 24 hour intervals and nuclei were counted in triplicate for each sample using a Beckman Coulter Cell counter. Cells treated with synthetic miR-34a showed a marked reduction in cell growth relative to premiR-negative control-treated groups in both SK-N-AS^luc ^and NB1691^luc ^cells (Figure 1C and D, respectively * p = 0.004). In order to extend findings previously reported by Welch *et al*., [6] in SK-N-AS cells, premiR-34a- treated NB1691^luc ^(3 × 10^5^) cells were isolated 48 and 72 hours post-transfection and analysed by flow cytometry. Cell cycle and Annexin-V analysis was carried out on all samples (n = 3) and data was normalised to premiR-negative control treated cells (Figure 1E and 1). Findings indicate that over expression of miR-34a leads to significant reduction in S phase progression (*p < 0.01), an increase in G0/G1 initiation (**p < 0.001) and a corresponding increase in apoptosis (*p < 0.01).

The *in vitro *effects of miR-34a ectopic over-expression were initially analysed on each of these cell lines. Mature miRNA-34a mimics (premiR-34a) or a negative control oligonucleotide (premiR-negative control) were transiently transfected into SK-N-AS^luc ^or NB1691^luc ^cells resulting in significantly enhanced expression of miR-34a. MiR-34a over-expression led to a significant reduction in mRNA levels of five experimentally validated miR-34a targets, *MYCN*, *BCL2*, *E2F1, E2F3 *and *CDC25A *in both cell lines; relative to premiR-negative control-treated cells (Figure 1A and 1B).

As expected, cell numbers were significantly reduced from 48 hours post-transfection relative to premiR-negative control-treated cells in both neuroblastoma cell lines (Figure 1C and 1D). Flow cytometry analysis of miR-34a transfected and premiR-negative control-treated NB1691^luc ^cells at both 48 and 72 hours post transfection indicated that miR-34a led to a significant reduction in the number of cells in S phase of the cell cycle (p < 0.01, biological replicates = 3), an increase in the percentage of cells in G0/G1 phase (p < 0.001, Figure 1E) and a substantial increase in cells entering apoptosis (p < 0.01, Figure 1F), consistent with reports by Welch *et al*., and Cole *et al*. involving SK-N-AS cells [6,8]. We conclude from these initial experiments that miR-34a over-expression has a pronounced anti-proliferative effect on NB1691^luc ^and SK-N-AS^luc ^cell lines cultured *in vitro*, consistent with prior publications [6,8].

### Alterations in cell signalling/phosphoprotein in response to miR-34 over-expression

Although miR-34a has been shown to directly target key genes such as *MYCN*, *E2F3 *and *BCL*2, the downstream effects of miR-34a over-expression on signal transduction pathways have not been investigated. We have quantified changes in the phosphorylation status of 8 proteins involved in various different signalling pathways including PI3K/AKT/mTOR signalling (p70 S6 kinase), RAS/RAF/MEK signalling (ERK1/2, STAT3, p70 S6 kinase, CREB), JAK/STAT signalling (STAT3, STAT5), heat shock or death receptor signalling (p38, JNK, IκB) and NF-κB signalling (IκB) following miR-34a ectopic over-expression in NB1691^luc ^cells using the MILLIPLEX MAP 8-plex Multi-Pathway Signalling Phosphoprotein Analysis kit, based on Luminex xMAP technology.

**Figure 2:**
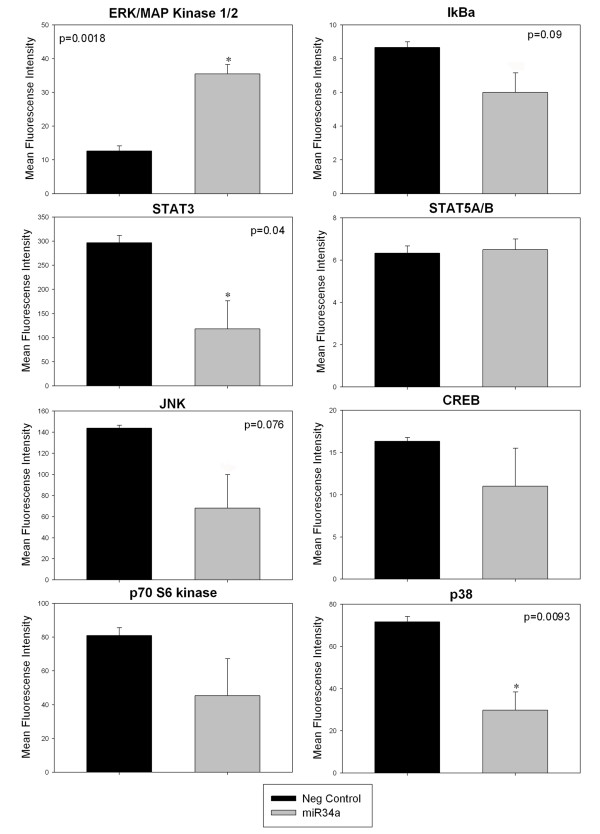
**Effects of miR-34a on cellular phosphoprotein activation in NB1691**^**luc **^**cells**. NB1691^luc ^(1 × 10^6^) cells were reverse transfected with premiR-34a (30 μM) or a premiR-negative control molecule. Protein lysates were isolated after 48 hours and 10 μg of total cell protein was analysed for phosphoprotein alterations using the MILLIPLEX MAP 8-Plex Multi-Pathway Signalling Phosphoprotein Kit (Millipore Corp.,). ERK/MAP Kinase 1/2 activation significantly increased in miR-34a treated cells relative to premiR-negative control treated NB1691^luc ^cells (p < 0.01 n = 3). Notably, activated STAT3, JNK and p38 levels tended towards a significant reduction relative to premiR-negative control- treated samples (p = 0.046, 0.07 and 0.009 respectively)

Over expression of miR-34a led to enhanced activation of ERK/MAP kinase 1/2 (p = 0.002, n = 3; Figure 2). Conversely, transfection of cells with synthetic miR-34a led to a significant reduction in STAT3 (p = 0.04) and p38 phosphorylation (p = 0.009) (Figure 2).

Additionally, c-Jun-N-terminal kinase (JNK) is a key regulator of apoptosis and, in miR-34a-treated NB1691^luc^cells, phosphorylated JNK levels are tending towards a significant reduction (p = 0.076) relative to activated JNK levels in control samples (Figure 2).

### miR-34a over-expression results in the down-regulation of *MAP3K9*

**Figure 3:**
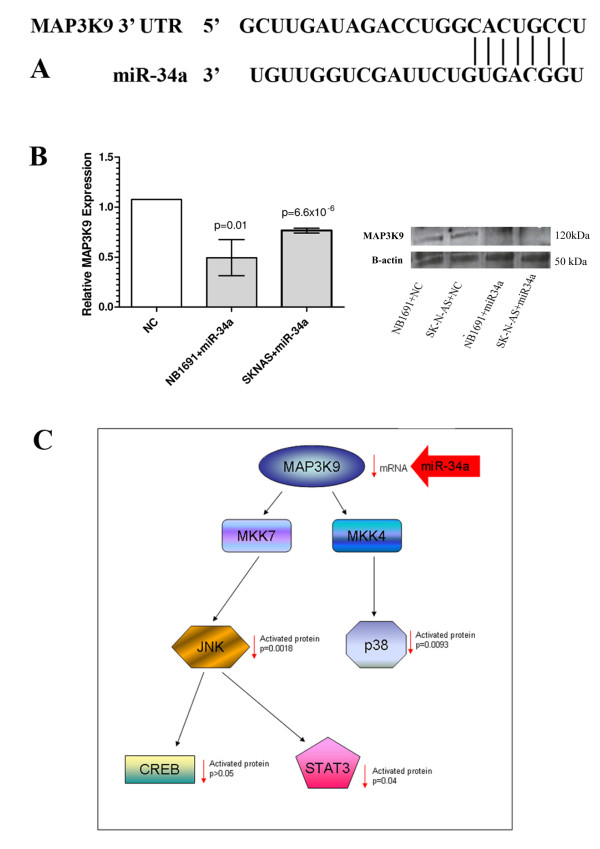
**Role of MAP3K9 in miR-34a-mediated apoptosis**. The 3'UTR of *MAP3K9 *was identified as a putative target of miR-34a with a 7-mer complementarity region (Figure 3A). Quantitative PCR and Western Blotting of miR-34a- treated NB1691^luc ^and SK-N-AS^luc ^cells was carried out and both *MAP3K9 *mRNA transcript and protein levels were shown to significantly decrease relative to premiR-negative control-treated samples in both cells lines analysed (n = 3). Figure 3C outlines the possible mechanism through which miR-34a might mediate cellular apoptosis, targeting and suppressing MAP3K9 expression, leading to downstream reduction in JNK, p38, CREB and STAT3 activated protein levels (verified by phosphoprotein analysis Figure 2)

Based upon the noted alterations in phosphoprotein activation levels, as discussed above, we examined the TargetScan miRNA prediction database [30] for potential kinases that might be direct targets of miR-34a that could account for these alterations. As illustrated in Figure 3A, the 3'UTR of *MAP3K9 (MLK1) *has a 7-mer complementarity region with the miR-34a seed region, leading us to examine the effects of miR-34a over expression on *MAP3K9 *mRNA transcripts and protein expression in NB1691^luc ^and SK-N-AS^luc ^cells. Notably, as shown in Figure 3B, the presence of miR-34a led to a significant reduction in *MAP3K9 *mRNA and protein expression in both neuroblastoma cell lines relative to premiR-negative control treated samples (n = 3). In order to validate that the 3' UTR of *MAP3K9 *is a direct target of miR-34a, a 1,140 base pair segment of the *MAP3K9 *3' UTR, inclusive of the miR-34a target site, was cloned into the 3' region of the luciferase gene in the PsiCheck2 vector (Psi/miR-34a, Additional File 2, Figure S2). In addition, a second construct was created with a 5 base pair mutation within the target seed site (Psi/miR34amut). As illustrated in Additional File 3, Figure S3, co-transfection of Psi/miR-34a with mature miR-34a mimics did not decrease luciferase activity relative to the negative control. Negative results for these experiments were obtained at different time points (24, 48 and 72 hrs) and with two cell lines (SK-N-AS and NB1691), indicating that either the miR-34a affect on *MAP3K9 *is not a direct effect, or that there is some conformational structural difference between the 3' UTR of the reporter versus the native 3' UTR, which inhibits miR-34a targeting of the reporter.

### MiR-34a has a highly significant tumor suppressor effect in an orthotopic mouse model of neuroblastoma

Although the role of miR-34a as a potential therapeutic *in vivo *has been studied in models of colon cancer [13], lung cancer [26] and glioblastoma[25] to date the ability of miR-34a to inhibit neuroblastoma cell growth has thus far only been investigated *in vitro*. Since several factors effecting tumor growth cannot be investigated through cell culture alone, the effects of miR-34a over-expression in an orthotopic murine model of neuroblastoma were investigated.

**Figure 4:**
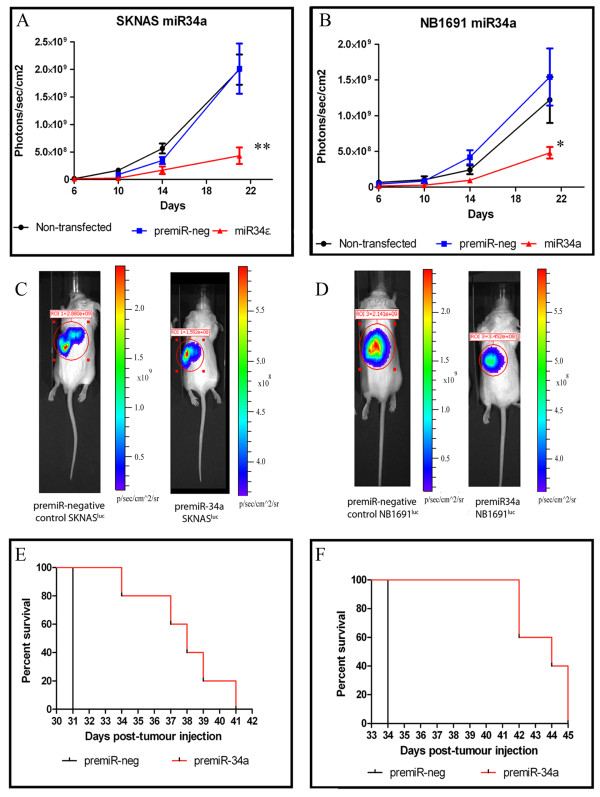
**Analysis of NB1691**^**luc **^**and SK-N-AS**^luc ^**xenografts**. MiR-34a or premiR-negative control-treated SK-N-AS^luc ^and NB1691^luc ^cells (4.4 × 10^5^), which were stably transfected with luciferase, were introduced into the retroperitoneal space of CB17-SCID immunocompromised mice (n = 4-7). Animals were administered an intraperitoneal injection of D-Luciferin (150 mg/kg) and, five minutes after substrate injection, the animals were imaged using an IVIS Imaging System 100 Series (Xenogen Corporation, Alameda, CA). Imaging was carried out at days 6, 10, 14 and 21 post-tumor cell inoculation and the data analysed and presented as the mean value for each cohort (photons/sec/cm^2^) ± SEM (*p < 0.001 and **p < 0.0001). The bioluminescent images above are representative of the photons/sec/cm^2 ^values obtained in premiR-negative control-treated SK-N-AS^luc ^(Figure 4A and C) and NB1691^luc ^(Figure 4B and D) animals compared to miR-34a-treated groups. Pre-treatment of both SK-N-AS^luc ^and NB1691^luc ^cells with synthetic miR-34a lead to significant reduction in tumor volume relative to premiR-negative control-treated cell tumors. Animals were sacrificed at moribundity and Mantle-Cox analysis was used to compare overall survival in xenograft cohorts (Figure 4E represents SK-N-AS^luc ^and Figure 4F represents the data obtained for NB1691^luc ^animals, p < 0.003 for both murine models).

Cells pre-transfected with miR-34a or the premiR-negative control were injected retroperitoneally into CB17-SCID mice (n = 4-6 per group) and tumor growth was detected through bioluminescence imaging facilitated by stable expression of the firefly luciferase gene in SK-N-AS^luc ^and NB1691^luc ^cells. Bioluminescent data indicates that expression of miR-34a in these murine models resulted in significant reduction in tumor volume up to 21 days post injection relative to premiR-negative control-treated groups (p < 0.001, Figure 4A-D). Control treated cells (premiR-negative control transfections) did not yield any significant variations in tumor volume relative to non-transfected cell induced tumors. Therefore, the effects which were noted can be considered to be a direct result of the introduction of miR-34a into the neuroblastoma cell, which were then subsequently used for tumor induction.

Bioluminescence data from tumor growth was collected up to 21 days post-tumor induction in each animal cohort. Subsequent to this time point, animals were sacrificed at moribundity. Notably, animals with miR-34a-treated tumors, both SK-N-AS^luc ^and NB1691^luc ^survived significantly longer than cohorts with premiR-negative control-treated tumors (Figure 4E and 4F, respectively; p < 0.003 in both tumor models).

## Discussion

This study has demonstrated that ectopic over-expression of miR-34a in the NB1691 cell line leads to alterations in phosphorylation levels of several key proteins involved with cell survival or apoptosis, including ERK, STAT3, P38 and JNK. These phosphorylation changes can be attributed to the down-regulation of *MAP3K9 *in response to miR-34a over-expression. The MAPKinase signal transduction pathway has been extensively studied with regards to its biochemical interactions and possibilities of therapeutic target identification (see Pearson for review [31]). MAP3K9 has been shown to phosphorylate both MKK7 and MKK4, amongst other target protein kinases (Figure 3C). Each of these kinases are, in turn, capable of phosphorylating and activating JNK and p38; respectively. Similarly, JNK can lead to activation of CREB and STAT3 transcription factors. With this pathway in mind, it is of interest to note that *MAP3K9 *suppression led to a significant reduction in JNK, p38 and STAT3 activated protein; each of which was verified by Multi-Pathway Signalling Phosphoprotein analysis. The identification of a possible pathway through which miR-34a over expression induces cell death provides a novel insight into the overall mechanism through which miR-34a-mediated apoptosis might occur in neuroblastoma.

ERK activation has been investigated with respect to its role as both a pro-survival and pro-apoptotic molecule; dependent upon cell type and duration of activation [32-34]. Notably, miR-34a induced cellular apoptosis of NB1691^luc ^neuroblastoma cells leads to a significant increase in phosphorylation of ERK1/2. Constitutive activation of STAT3 has been correlated to poor prognosis in both colorectal cancer [35] and non-small cell lung cancer [36]. STAT3 inactivation in neuroblastoma cell lines has been observed in response to various treatments such as Sorafenib [37] and Curcurbitacin [38]. In this context, miR-34a over-expression in neuroblastoma cells and the subsequent inactivation of the STAT3 pathway appears to be one of the potential mechanisms through which miR-34a may exert its apoptotic effect in these cells.

P38 mitogen-activated protein kinases are responsive to stress stimuli and involved in cell differentiation and apoptosis. Studies of pathway activation in lung cancer led to the identification of activated p38 in all tumor samples analysed (n = 19); suggesting a role for activated p38 in tumor progression and maintenance [39]. Concomitant with this theory is the noted reduction in p38 activation in NB1691^luc ^cells in response to miR-34a over-expression, a treatment which induces cellular apoptosis in neuroblastoma cells. Activated JNK has been proven to be both pro-and anti-apoptotic in a variety of cell lines [40]. Notably, inhibition of activated JNK led to apoptosis in lung carcinoma cells supporting findings in NB1691^luc ^cells where miR-34a-induced apoptosis reduces JNK activation [41,42]. In the context of the neuroblastoma cell line SK-N-SH, it is of interest that inhibition of JNK basal levels through either a JNK specific inhibitor or through siRNA mediated knock-down results in increased P53 protein [43]. Thus, given that P53 directly activates miR-34a transcription [14,20-22], it is possible that miR-34a enhances its own activation indirectly leading to dephosphorylation and inactivation of JNK.

Although luciferase reporter assays failed to demonstrate direct targeting of *MAP3K9 *by miR-34a, in our opinion, these negative results do not rule out the possibility of direct targeting, as conformational differences between the luciferase 3' UTR and that of the endogenous *MAP3K9 *could have affected targeting. From a functional standpoint, down-regulation of *MAP3K9 *by miR-34a either through direct targeting or an alternative secondary mechanism would be expected to have the same phenotypic consequences.

Identification of miR34a as a potent tumor suppressor molecule of neuroblastoma *in vivo *is a highly significant finding with respect to the development of potential therapeutics for this disease. Current therapies for high risk neuroblastoma include chemo- and radiation-therapy in an attempt to hinder tumor relapse. Identification of miRNA-mediated gene therapies for neuroblastoma provides a potential alternative with respect to treatment which may circumvent current issues including chemotherapeutic drug resistance in certain tumors and adverse drug treatment side effects. Targeted therapeutics utilising the efficacy of miR34a in this disease state is a novel area of research in terms of neuroblastoma tumor treatment.

## Conclusions

The role of miRNAs in mediating critical cellular processes is an emerging field in cancer genetics. Dysregulation, enhanced expression and selective inhibition of miRNAs has improved scientific understanding of the functional role which these regulatory molecules play in cancer progression and patient prognosis. MiR-34a was the first miRNA identified as a putative tumor suppressor in neuroblastoma through its direct targeting of transcription factors and other genes essential for cellular proliferation. Here we identify, for the first time, the efficacy of miR-34a in retarding neuroblastoma tumor growth *in vivo *in both *MYCN *amplified (NB1691^luc^) and non-*MYCN *amplified (SK-N-AS^luc^) neuroblastoma xenografts; and also propose a potential mechanism through which this might occur. The success which transient pre-treatment of these cells with miR-34a has on tumor growth provides rationale for further investigation of the effects of miR-34a in pre-established tumors *in vivo*; a task which is currently being undertaken by our research team.

## Abbreviations

UTR: (untranslated region); qPCR: (quantitative PCR); miRNA: (micro RNA); *MYCN*: (myc myelocytomatosis viral related oncogene, neuroblastoma derived); *BCL-2*: (B-cell lymphoma 2); *SIRT1*: (silent information regulator 1); *SFRP1*: (secreted frizzled-related protein 1); *CAMTA1*: (calmodulin-binding transcription activator 1); *JAG1*: (Jagged 1); *CCND1*: (cyclin D1); *CDK6*: (cyclin-dependent kinase 6).

## Competing interests

The authors declare that they have no competing interests.

## Authors' contributions

AT and LT carried out all laboratory work pertaining to this study, participated in its design and drafted the final manuscript. PGB and LA carried out arrayCGH analysis of Kelly, SK-N-AS and SK-N-AS^luc ^cell lines. AMD provided expertise on *in vivo *neuroblastoma modelling, access to animal facilities in St. Jude Children's Research Hospital, Memphis, TN and manuscript editing. RLS conceived the study, and participated in its design and coordination and helped to draft the manuscript. All authors read and approved the final manuscript.

## Pre-publication history

The pre-publication history for this paper can be accessed here:

http://www.biomedcentral.com/1471-2407/11/33/prepub

## Supplementary Material

Additional File 1 **Figure S1. Whole genome DNA copy number plots of neuroblastoma cell lines**. Array-CGH profiles for *MYCN *amplified cell lines (A) Kelly and (B) NB1619^luc ^as well as 11q- cell lines (C) SK-N-AS^luc ^and (D) SK-N-AS. The y-axis represents the log_2 _fluorescent ratios of cell line (Cy3) versus a reference control (Cy5). Chromosomes are plotted across the x-axis from chromosome 1 to chromosome Y. Click here for file

Additional File 2 **Figure S2. Segment of MAP3K9 3' UTR cloned into the PsiCheck2 luciferase reporter plasmid**. (A) Wild type sequence with miR-34a seed region highlighted in yellow. (B) Mutated sequence with mutated sites within the miR-34a seed region highlighted in green. Click here for file

Additional File 3 **Figure S3. Luciferase reporter assays**. SK-N-AS (A, C and E) and NB1691 (B, D and F) cells were transiently transfected with Psi/miR34a or Psi/miR34amut plasmid in conjunction with premiR34a or premiR-negative control molecules. Direct targeting, through luciferase activity analysis relative to co-expressed renilla activity, could not be validated in either cell lines at the three time points assessed. Click here for file
