# Supplementary figures and images for: MicroRNA-34a is a potent tumor suppressor molecule in vivo in neuroblastoma
Source: BMC Cancer. 2011 Jan 25;11:33. doi: 10.1186/1471-2407-11-33 (PMC3038978; doi:10.1186/1471-2407-11-33)

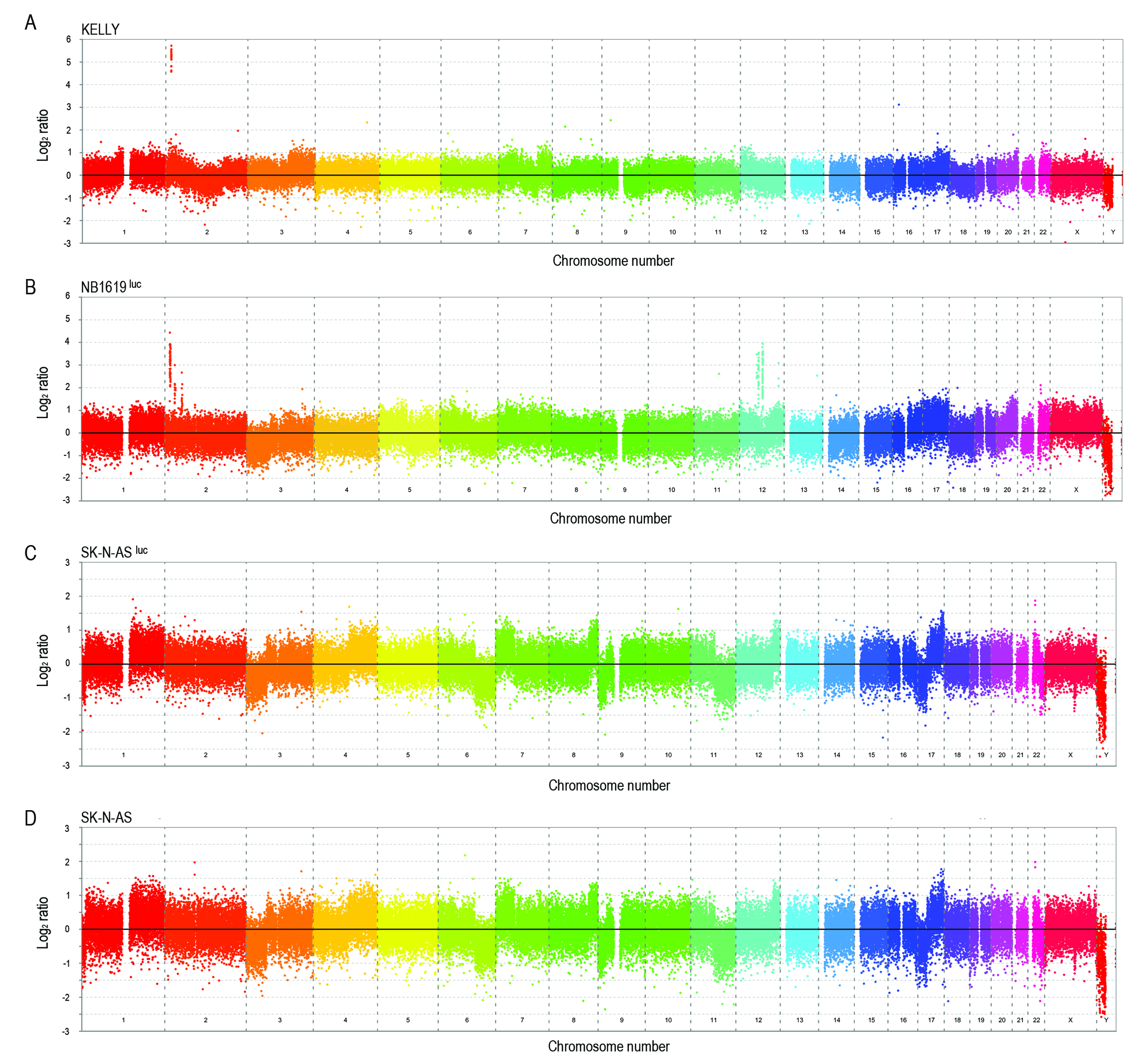

Supplement: Additional File 1 — Figure S1. Whole genome DNA copy number plots of neuroblastoma cell lines. Array-CGH profiles for MYCN amplified cell lines (A) Kelly and (B) NB1619luc as well as 11q- cell lines (C) SK-N-ASluc and (D) SK-N-AS. The y-axis represents the log2 fluorescent ratios of cell line (Cy3) versus a reference control (Cy5). Chromosomes are plotted across the x-axis from chromosome 1 to chromosome Y. [file 1471-2407-11-33-S1.TIFF]

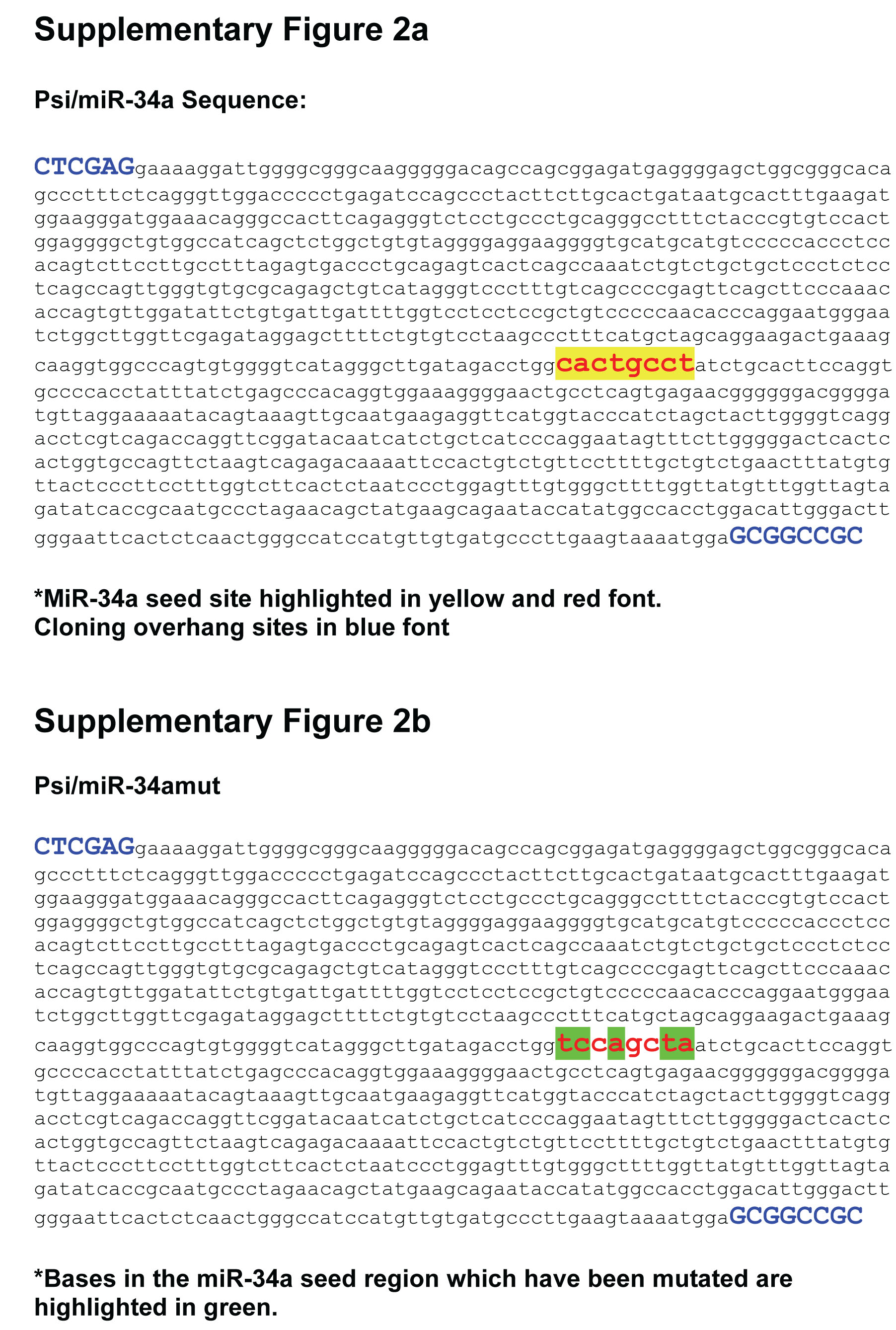

Supplement: Additional File 2 — Figure S2. Segment of MAP3K9 3' UTR cloned into the PsiCheck2 luciferase reporter plasmid. (A) Wild type sequence with miR-34a seed region highlighted in yellow. (B) Mutated sequence with mutated sites within the miR-34a seed region highlighted in green. [file 1471-2407-11-33-S2.JPEG]

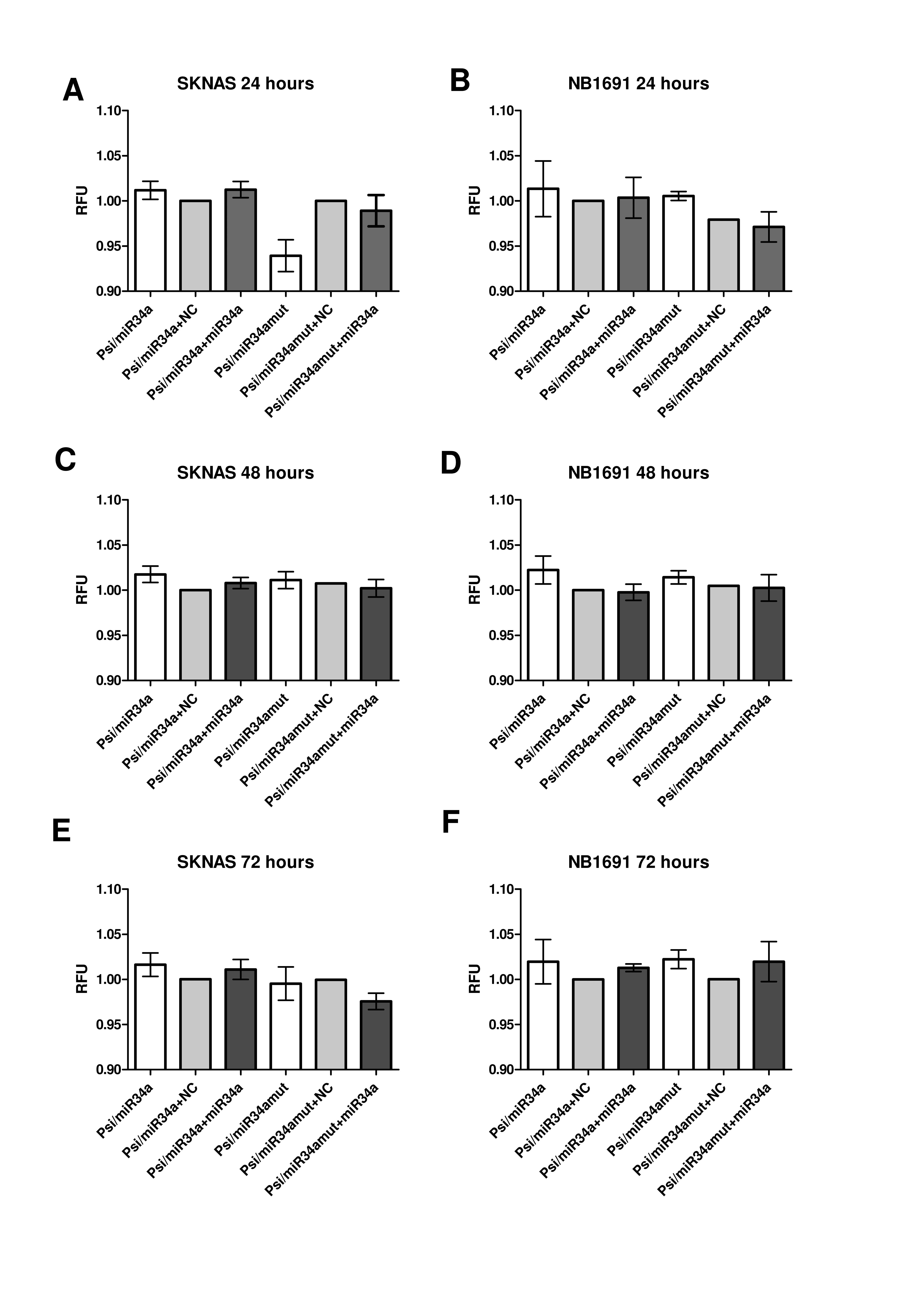

Supplement: Additional File 3 — Figure S3. Luciferase reporter assays. SK-N-AS (A, C and E) and NB1691 (B, D and F) cells were transiently transfected with Psi/miR34a or Psi/miR34amut plasmid in conjunction with premiR34a or premiR-negative control molecules. Direct targeting, through luciferase activity analysis relative to co-expressed renilla activity, could not be validated in either cell lines at the three time points assessed. [file 1471-2407-11-33-S3.JPEG]
